# CaviDB: a database of cavities and their features in the structural and conformational space of proteins

**DOI:** 10.1093/database/baad010

**Published:** 2023-05-10

**Authors:** Ana Julia Velez Rueda, Franco Leonardo Bulgarelli, Nicolás Palopoli, Gustavo Parisi

**Affiliations:** Departamento de Ciencia y Tecnologia, Universidad Nacional de Quilmes, Roque Saenz Pena 182, Bernal B1876BXD, Argentina; Departamento de Ciencia y Tecnologia, Universidad Nacional de Quilmes, Roque Saenz Pena 182, Bernal B1876BXD, Argentina; Departamento de Ciencia y Tecnologia, Universidad Nacional de Quilmes, Roque Saenz Pena 182, Bernal B1876BXD, Argentina; Departamento de Ciencia y Tecnologia, Universidad Nacional de Quilmes, Roque Saenz Pena 182, Bernal B1876BXD, Argentina

## Abstract

Proteins are the structural, functional and evolutionary units of cells. On their surface, proteins are shaped into numerous depressions and protrusions that provide unique microenvironments for ligand binding and catalysis. The dynamics, size and chemical properties of these cavities are essential for a mechanistic understanding of protein function. Here, we present CaviDB, a novel database of cavities and their features in known protein structures. It integrates the results of commonly used cavity detection software with protein features derived from sequence, structural and functional analyses. Each protein in CaviDB is linked to its corresponding conformers, which also facilitates the study of conformational changes in cavities. Our initial release includes ∼927 773 distinct proteins, as well as the characterization of 36 136 869 cavities, of which 1 147 034 were predicted to be drug targets. The structural focus of CaviDB provides the ability to compare cavities and their properties from different conformational states of the protein. CaviDB not only aims to provide a comprehensive database that can be used for various aspects of drug design and discovery but also contributes to a better understanding of the fundamentals of protein structure–function relationships. With its unique approach, CaviDB represents an indispensable resource for the large community of bioinformaticians in particular and biologists in general.

**Database URL** https://www.cavidb.org

## Introduction

Proteins are the functional, structural and evolutionary units of cells. They consist of chains of amino acids that interact in complex and highly interconnected networks. On their surface, proteins are shaped into numerous cavities and protrusions that provide unique microenvironments for ligand binding or catalysis (1). The dynamic of these cavities are fundamental for understanding protein function, and their variations can explain changes in protein activity (2–5). Protein movements, even the smallest, can affect cavity architecture (6, 7). On different time scales, the movements are required not only to bind the substrate or determine its affinity constant but also to allow ligand transit from the surface to the active site (8).

The size and geometry of the cavities, as well as their accessibility, have proven useful in making predictions about protein–protein interactions, protein pharmacology and binding specificity (9–11). For example, physicochemical properties of the cavities such as their charge or hydrophobicity can also be used to predict the binding probability of specific ligands (12, 13). Residues are known to shift their p*K*_a_ values based on various structural and environmental features (14, 15), which favors various biological activities (16, 17). In addition, it has been shown that the shape and location of cavities in proximity to each other can determine their relative flexibility and influence their catalytic and binding promiscuity (4, 11, 18).

Functional cavities are generally located within protein domains, which are evolutionarily conserved protein regions with specific stability, function and dynamics. The biological activity of individual cavities is not always correlated with domain function, and the conservation of cavities may exceed that of a particular domain family. Therefore, knowledge of domain activity is not sufficient to fully understand protein function, and the integrative characterization of all domains and their cavities may be a better approach (19).

Here, we present CaviDB (https://www.cavidb.org/), an interactive online database that integrates the results of commonly used cavity detection software with protein features retrieved from sequence, structural and functional analyses. CaviDB implements established cavity detection methods (20, 21) that allow local structural characterization but is also useful to understand protein anatomy and function on a global scale (22). Our database allows users to explore protein dynamics through an easy-to-use interface that facilitates the comparison of the properties of protein conformers and their predicted cavities. CaviDB provides structural data on every known protein structure available in the Protein Data Bank (23) and on the protein structure predictions of entire proteomes from model organisms available in the AlphaFold database (24). Our goal is to provide a comprehensive resource for use in various biotechnological applications, such as drug development and discovery, but also for a better understanding of the fundamentals of the relationship between protein structure and function.

## Materials and methods

### Cavity prediction and categorization

CaviDB provides users with structural and sequential features to characterize protein cavities. Cavity predictions were performed using the widely used Fpocket software (25) with default settings for all entries in the Protein Data Bank (26, 27) and all the AlphaFold database entries (28). We retrieved and annotated all properties (Supplementary Table S1) associated with each cavity and all its lining residues. The cavity was considered to be druggable if it had an affectability value >0.5, as suggested in previous work (20).

### Cavities features’ calculation

To provide users with information on possible activated cavities, we estimated the p*K*_a_ values (at pH = 7) of the ionizable residues and their shifts (p*K*_a_ predicted − p*K*_a_ expected) using PROPKA (29). The net p*K*_a_ shift values per cavity were calculated as the sum of all absolute p*K*_a_ shifts of each ionizable residue belonging to a cavity.

Using PROPKA, we also retrieved data on inter-residue contacts per site to annotate the contacts of the cavities as side-chain hydrogen bonds, backbone hydrogen bonds and coulombic bonds. We created a network of cavities that have at least one contact between the same sites, which can be displayed as an interactive diagram. The binding energy heat maps show the contacts between cavities by calculating the sum of the absolute binding energies between the residues that make contact in the corresponding pair of cavities and rendering colored squares.

Different physicochemical properties per site were calculated using Classification of Intrinsically Disordered Ensemble Regions (30), modlAMP (31) and Biopython (32) and assigned to each cavity as the mean values of the properties of its residues.

### Global protein features’ calculation and annotation

Global protein features were calculated as described in the previous section. Each Protein Data Bank entry (PDB) chain or AlphaFold model was annotated via Structure Integration with Function, Taxonomy and Sequence (33) with identifiers of relevant biological databases such as CATH (34) and Pfam (35) to facilitate subsequent analysis by users.

### Conformational comparisons

For the conformers’ cavities comparisons, we used the PDBSWS—PDB/UniProt Mapping (36). This database maps PDB residues to residues in UniProtKB (Swiss-Prot and TrEMBL) entries (37), consequently allowing the precise comparison between cavities of different entries.

### Web application overview

A responsive web interface was developed to display the data stored in a non-relational database, allowing easier navigation and visualization of the database contents on different devices. The web application was implemented in HTML, CSS, Ruby (on Rails) and JavaScript (using NodeJS).

**Figure 1.**
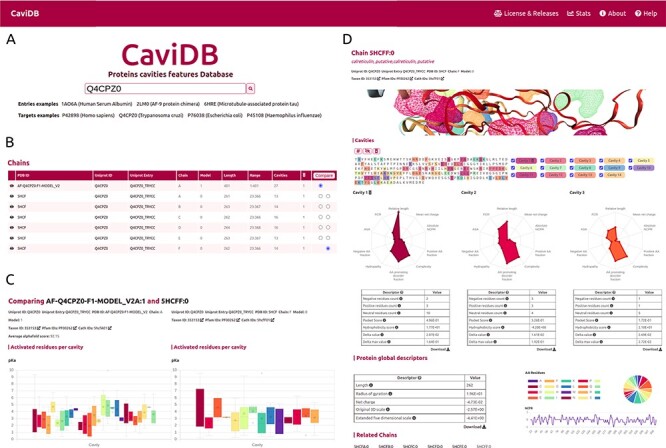
Overview of the CaviDB web application. (A) CaviDB search allows users to search for a specific PDB or UniProt identifier. A selector is also provided to focus the search on AlphaFold models. (B, C) Cavity dynamics can be explored using the comparison tool provided by CaviDB, where predicted cavities and their features can be selected and displayed for different protein conformations simultaneously. (D) Schematic example of chain feature display. The information of each entry is divided into two main sections, one containing the general cavity descriptors (top) and the other containing the global protein descriptors (bottom).

The first step for running CaviDB is to provide a valid PDB or UniProt ID. The web server automatically loads all chains related to the search, as well as their general data, including their length, the number of predicted cavities and relevant cross-reference identifiers (Figure 1A and B). The search can be filtered using the AlphaFold selector if the user is only interested in these sorts of entries. The features obtained for each entry are organized into two main sections describing the general cavity descriptors, including an interactive display for visualizing the cavities, a network representation of the interactions and cavities including activated residues with p*K*_a_ shifts and the global protein descriptors (Figure 1D).

CaviDB allows users to explore the conformational diversity of proteins and its impact on cavity dynamics by providing a conformational comparator (Figure 1B) that displays a comparison page with the listed cavities for each chain and, when selected, their properties and residues.

## Results and Test cases

### Globular protein test case

Promiscuous proteins are a breaking point in the “structure–function” paradigm and the concept of biological specificity (38, 39). Promiscuous protein behavior presents both challenges and opportunities for drug discovery programs and has been explored as a strategy for drug repurposing (40–42).

Human serum albumin (HSA) is the major protein in plasma, binds multiple ligands (43) and has recently emerged as a very important drug carrier (44, 45). It has several high-affinity binding sites, but most drugs and ligands bind to the so-called sites I (from Met 1 to Asn 111) and II (from Gln 196 to Pro 303) (46). HSA has previously been described not only as a transport protein but also as a promiscuous enzyme possibly related to salicylic acid metabolism and side effects (18, 47–50).

**Figure 2.**
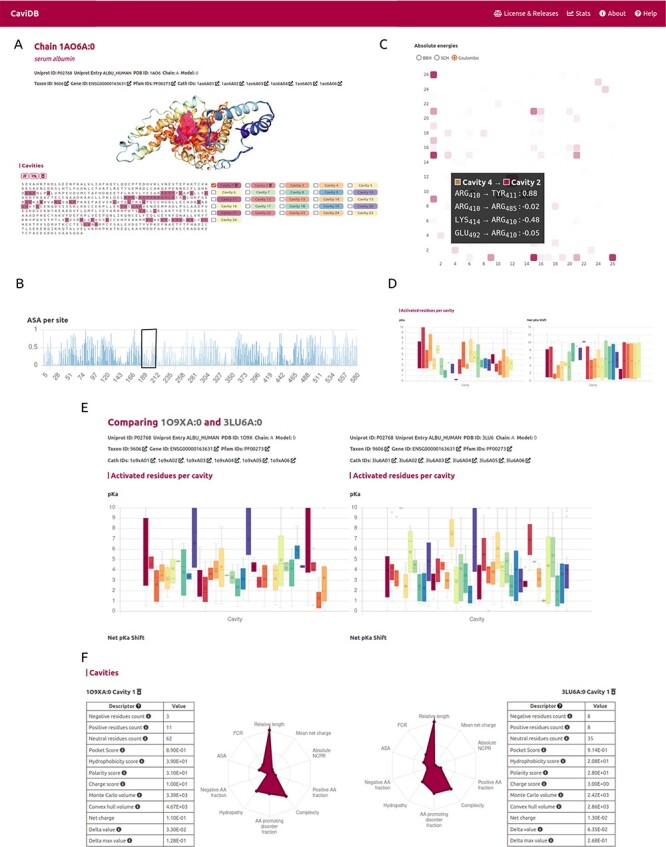
CaviDB display of HSA (PDB ID: 1AO6, chain: A, model: 0). (A) Display of protein and residues per cavity, with Cavities 1 and 2 highlighted in the sequence (magenta and pink, respectively). (B) Bar plot of normalized accessible surface area per site. A box highlights a local minimum of ASA in the vicinity of Lys199. (C) Interactive heat map display of all possible pairwise contacts established between residues in different cavities. A popup window provides details on the absolute energies of the selected interaction. Heat map colors correspond to the number of interactions per cavity, the larger the interaction number is, the darker the color. (D) Boxplot distribution of p*K*_a_ values (left) and p*K*_a_ shifts (right) per residue in each detected cavity. (E) Comparison of HSA conformers with high RMSD. The panel shows important changes in acid–base properties in Cavity 1 resulting from the conformational changes (mean p*K*_a_ = 3.99 in 3LU6_A vs. mean p*K*_a_ = 5.16 in 1O9X_A), along with changes in other physicochemical features such as the number of charged amino acids, hydrophobicity and charge of residues (F).

It has been proposed that the basis for the great ability of albumins to catalyze various reactions lies in the existence of activated amino acids with abnormal p*K*_a_ in the hydrophobic cavity of the AII binding site, which creates a microenvironment favorable for catalysis (18, 51). As shown by the per-site solvent accessibility plot (ASA) generated by CaviDB for the 1AO6A:0 entry, there is a local minimum around Lys199 and Arg222 (see Figure 2B), a region described as important for catalysis (50, 52). These important catalytic residues are located in the AII binding site identified by CaviDB as the largest cavity (Cavity 1) in the entry’s star plot with the highest relative length parameter (equal to 1), also showing a large number of contacts between cavities and the presence of activating residues. Residues Lys199 and Arg222 show essential p*K*_a_ shift in order to sustain the catalytic activity, showing abnormally acidic properties (Lys, 199, ∼7.51 and Arg, 222, ∼9.49 (18). Using the information deposited in the CoDNaS database (53), we found the pair of HSA conformers showing the maximum conformational diversity (pairs 3LU6_A and 1O9X_A with an Root-mean-square deviation  = 6.27 Å). Using this information and the comparison capability of conformers in CaviDB, it is also possible to compare the change in some cavity features. It is then possible to observe differences in the acid–base properties of Cavity 1, such as in the mean p*K*_a_ of Cavity 1 (Figure 2E) and changes in charge and hydrophobicity (Figure 2F).

A second cavity (Cavity 2) containing residues Arg410 and Tyr411, previously described as part of the catalytic active site, was also identified (47) (Figure 2). In addition, Cavity 2 contains tyrosine 411 and arginine 410 (belonging to Cavity 4), two residues that have been shown to be important for the esterase-like activity of the protein (52) and that interact with each other through coulombic forces (Figure 2C). In this way, CaviDB gathers important information that provides a mechanistic explanation for the promiscuous behavior of HSA as described previously (18, 54).

### Using AlphaFold models for better predictions

**Figure 3.**
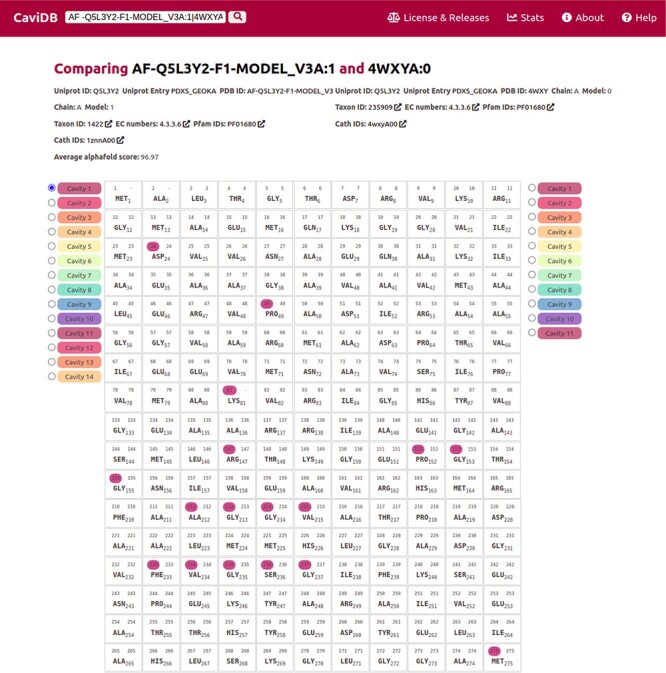
Comparison of the presence of cavities in PLP synthase conformers (UniProt ID Q5L3Y2). Using the expression AF-Q5L3Y2-F1-MODEL_V3A:1|4WXYA:0 to search in CaviDB allows comparing the presence of cavities in both selected conformers. It can be seen that the AlphaFold model contains a biologically relevant cavity (Cavity 1) that contains the key residues described in the bibliography (56). This cavity is absent in other conformers due to the presence of disordered regions.

The recent breakthrough of AlphaFold in predicting 3D models provides new opportunities for exploring protein–structure relationships. In CaviDB, we have included 1 029 746 AlphaFold models, but we plan to include all recently released models in future upgrades (https://alphafold.ebi.ac.uk/). Recently, AlphaFold models were found to correctly predict some of the native conformations of protein ensembles (55). In some cases, high-quality models could help to assess the functional implications of cavities. Pyridoxal 5ʹ-phosphate (PLP) synthase (PLPS) is a biosynthetic pathway enzyme that produces PLP from glutamine, ribose 5-phosphate and glyceraldehyde 3-phosphate. The native state of PLP synthase consists of 12 synthase and 12 glutaminase subunits, and its chemical mechanism has already been described (56). The active site contains active Lys81 and Asp24 (57, 58). In some conformers of the enzyme, this active site is open, which is due to the presence of a disordered region over the binding site (residues 49–56) (58). When known PLPS conformers are tested for the presence of cavities in CaviDB (using UniProt ID Q5L3Y2 or PDBs 4wy0 and 4wxz), no cavities containing biologically active residues are found. This is likely due to the fact that the binding site is open in these experimental structures. However, when AlphaFold models of PLPs are considered, a new cavity is discovered that contains the biologically relevant residues (Figure 3.) In this sense, the use of high-quality AlphaFold models could help in the estimation of cavities and their potential biological role.

### The advantages of CaviDB over existing services

CaviDB has a total count of 927 773 distinct proteins, with 740 140 conformers from the PDB and 1 029 746 from the AlphaFold database. It annotates proteins from 14 871 species representing 10 181 Pfam families. With the number of entries in our first release, we were able to characterize a total of 36 136 869 cavities, of which 1 147 034 are druggable. Since CaviDB provides gene IDs and Ensembl IDs, the data of each entry can be easily linked to metabolic pathways and evolutionary information in which each protein might be involved. Moreover, CaviDB is the first repository of information regarding protein cavities that explicitly considers the state-of-the-art AlphaFold models as targets for cavity discovery. Of AlphaFold models in CaviDB, 8042% are above a pLDDT score of 70, offering in this way a substantial amount of 3D models with a considerable level of predicted quality. Furthermore, this is also especially interesting for intrinsically disordered proteins or proteins with flexible regions, in which much of the structural information of biological relevance is not observable to experimental techniques such as X-ray crystallography. There are many tools focused on protein structural characterization and cavity prediction (59, 60), such as CavitySpace, a library focused on cavities in human proteins predicted by AlphaFold, or CavityPlus, a web server for cavity detection. In addition, the number of predicted 3D models is growing very rapidly, characterizing almost the entire known sequence space (https://alphafold.ebi.ac.uk/) (24) and providing unprecedented opportunities to study the structure–function relationship of proteins. However, as we have shown, CaviDB is not only a tool for determining the properties of protein cavities and their dynamics in a large number of different species and proteins but also provides a simple and accessible way to analyze structural data.

## Discussion

Identification of binding cavities is critical for understanding the relationship between protein structure and function and is a crucial step for drug design (13, 59, 61, 62). Since conformational diversity is a key concept for understanding protein biology, CaviDB provides not only a freely accessible, comprehensive database of features of proteins and their cavities but also a simple and user-friendly tool for analyzing the data with a dynamic perspective at multiple levels.

## Supplementary Material

baad010_Supp
