# Supplementary material for: CaviDB: a database of cavities and their features in the structural and conformational space of proteins
Source: Database (Oxford). 2023 May 10;2023:baad010. doi: 10.1093/database/baad010 (PMC10171230; doi:10.1093/database/baad010)
Supplement: baad010_Supp [file baad010_supp.zip › suppl_data/[Database] Supplementary_material.docx]

**Supplementary Tables**

Supplementary Table 1. CaviDB global proteins and cavities descriptors, calculated using CIDER [(1)](https://sciwheel.com/work/citation?ids=2984117&pre=&suf=&sa=0), modlAMP [(2)](https://sciwheel.com/work/citation?ids=6001910&pre=&suf=&sa=0), Biopython [(3)](https://sciwheel.com/work/citation?ids=1341590&pre=&suf=&sa=0), PROPKA [(4)](https://sciwheel.com/work/citation?ids=953515&pre=&suf=&sa=0), and Fpocket [(5)](https://sciwheel.com/work/citation?ids=106566&pre=&suf=&sa=0).

| **Descriptor** | **Value** |
| --- | --- |
| Negative Residues Count | [CIDER] Get the number of negatively charged residues in the sequence (D/E) |
| Positive Residues Count | [CIDER] Get the number of positively charged residues in the sequence (R/K) |
| Neutral Residues Count | [CIDER] Get the number of neutral amino acids |
| Pocket Score | [Fpocket] Internal parameter |
| Hydrophobicity score | [Fpocket] Based on a residue based hydrophobicity scale published by [(6)](https://sciwheel.com/work/citation?ids=3104281&pre=&suf=&sa=0) |
| Polarity score | [Fpocket] Hydrophilicity character of a binding pocket |
| Charge score | [Fpocket] Charge of each amino acid in the binding site is tracked |
| Monte Carlo volume | [Fpocket] Feature based on Monte­-Carlo algorithm calculations, full volume occupied by all alpha sphere in a given pocket |
| Convex hull volume | [Fpocket] This data resumes relative volume of different amino acids |
| Net charge | [CIDER] Get the net charge per residue of the sequence NCPR |
| Delta value | [CIDER] Returns the delta value of the sequence, as defined when calculating kapp |
| Delta Max Value | [CIDER] Returns the maximum possible delta value (delta-max) for a sequence of this composition |
| Length | Sum of sequence characters |
| Count Neutral Res | [CIDER] Get the number of neutral amino acids |
| Delta value | [CIDER] Returns the delta value of the sequence, as defined when calculating kapp |
| Delta Max Value | [CIDER] Returns the maximum possible delta value (delta-max) for a sequence of this composition |
| Mean net charge | [CIDER] Get the absolute mean net charge of the sequence (pH=7) |
| Fraction of aminoacids expanding residues | [CIDER] Get the fraction of residues which are predicted to contribute to chain expansion (E/D/R/K/P) |
| Fraction of aminoacids promoting disorder residues | [CIDER] Get the fraction of residues predicted to be ‘disorder promoting’ |
| Aliphatic index | [MODLAMP] Method to calculate the aliphatic index of every sequence in the attribute sequences. |
| Aromaticity | [MODLAMP] Method to calculate the aromaticity of every sequence in the attribute sequences. |
| Predicted charge | [MODLAMP] Method to overall charge of the sequence (based on Bjellqvist method) |
| Charge density | [MODLAMP] Method to calculate the charge density (charge / MW) of the sequences |
| Hydrophobic ratio | [MODLAMP] Relative frequency of the amino acids A, C, F, I, L, M & V |
| Instability index | [MODLAMP] The instability index is a prediction of protein stability based on the amino acid composition ([1] K. Guruprasad, B. V Reddy, M. W. Pandit, Protein Eng. 1990, 4, 155–161.) |
| Isoelectric point | [MODLAMP] The isoelectric point of the sequence, based on the pK scale is extracted from CRC Handbook of Chemistry and Physics, 96th ed |
| Selectivity Index | [MODLAMP] Sequence selectivity index scale for helical antimicrobial |
| Argos hydrophobicity scale | [MODLAMP] Sequence Argos hydrophobicity |
| Bulkiness | [MODLAMP] Sequence bulkiness, based on amino acid side chain bulkiness scale |
| Eisenberg hydrophobicity scale | [MODLAMP] Sequence hydrophobicity based on Eisenberg consensus |
| Grantham side-chain descriptor | [MODLAMP] Sequence side chain composition, polarity and molecular volume |
| pepArc | [MODLAMP] Pharmacophoric feature scale, dimensions are: hydrophobicity, polarity, positive charge, negative charge, proline |
| T-scale | [MODLAMP] A PCA derived scale based on amino acid side chain properties calculated with 6 different probes of the GRID program |
| Extended  five-dimensional scale | [MODLAMP] The extended five dimensional Z-scale (z5) |
| Original 3D scale | [MODLAMP] The original three-dimensional Z-scale (z3) |
| Intercavities  Contacts | [Propka] BBH, SCH, and Coulombic binding energies between residues |
| Ionizable residues pKa | [Propka] Ionizable residues pKa |
|  |  |

References

[1. Holehouse, A. S., Das, R. K., Ahad, J. N., et al. (2017) CIDER: Resources to Analyze Sequence-Ensemble Relationships of Intrinsically Disordered Proteins. *Biophys. J.*, **112**, 16–21.](https://sciwheel.com/work/bibliography/2984117)

[2. Müller, A. T., Gabernet, G., Hiss, J. A., et al. (2017) modlAMP: Python for antimicrobial peptides. *Bioinformatics*, **33**, 2753–2755.](https://sciwheel.com/work/bibliography/6001910)

[3. Chapman, B. and Chang, J. (2000) Biopython. *SIGBIO Newsl.*, **20**, 15–19.](https://sciwheel.com/work/bibliography/1341590)

[4. Olsson, M. H. M., Søndergaard, C. R., Rostkowski, M., et al. (2011) PROPKA3: Consistent Treatment of Internal and Surface Residues in Empirical pK Predictions. *J. Chem. Theory Comput.*, **7**, 525–537.](https://sciwheel.com/work/bibliography/953515)

[5. Le Guilloux, V., Schmidtke, P. and Tuffery, P. (2009) Fpocket: an open source platform for ligand pocket detection. *BMC Bioinformatics*, **10**, 168.](https://sciwheel.com/work/bibliography/106566)

[6. Monera, O. D., Sereda, T. J., Zhou, N. E., et al. (1995) Relationship of sidechain hydrophobicity and alpha-helical propensity on the stability of the single-stranded amphipathic alpha-helix. *J. Pept. Sci.*, **1**, 319–329.](https://sciwheel.com/work/bibliography/3104281)
